# Supplementary material for: Hybrid AI-enhanced lightning flash prediction in the medium-range forecast horizon
Source: Nat Commun. 2024 Feb 8;15:1188. doi: 10.1038/s41467-024-44697-2 (PMC10853497; doi:10.1038/s41467-024-44697-2)
Supplement: Supplementary file 2 — Reporting Summary [file 41467_2024_44697_MOESM2_ESM.pdf]

nature portfolio

Corresponding author(s): Andrea Mazzino

Last updated by author(s): 06/12/2023

Reporting Summary

Nature Portfolio wishes to improve the reproducibility of the work that we publish. This form provides structure for consistency and transparency in reporting. For further information on Nature Portfolio policies, see our Editorial Policies and the Editorial Policy Checklist.

Statistics

For all statistical analyses, confirm that the following items are present in the figure legend, table legend, main text, or Methods section.

n/a	Confirmed	
☐	☒	The exact sample size (*n*) for each experimental group/condition, given as a discrete number and unit of measurement
☐	☒	A statement on whether measurements were taken from distinct samples or whether the same sample was measured repeatedly
☒	☐	The statistical test(s) used AND whether they are one- or two-sided<br>*Only common tests should be described solely by name; describe more complex techniques in the Methods section.*
☐	☒	A description of all covariates tested
☒	☐	A description of any assumptions or corrections, such as tests of normality and adjustment for multiple comparisons
☐	☒	A full description of the statistical parameters including central tendency (e.g. means) or other basic estimates (e.g. regression coefficient) AND variation (e.g. standard deviation) or associated estimates of uncertainty (e.g. confidence intervals)
☒	☐	For null hypothesis testing, the test statistic (e.g. *F*, *t*, *r*) with confidence intervals, effect sizes, degrees of freedom and *P* value noted<br>*Give P values as exact values whenever suitable.*
☒	☐	For Bayesian analysis, information on the choice of priors and Markov chain Monte Carlo settings
☒	☐	For hierarchical and complex designs, identification of the appropriate level for tests and full reporting of outcomes
☒	☐	Estimates of effect sizes (e.g. Cohen's *d*, Pearson's *r*), indicating how they were calculated

Our web collection on statistics for biologists contains articles on many of the points above.

Software and code

Policy information about availability of computer code

Data collection	Data have been managed by Python lenguage. It is a versatile and high-level programming language known for its simplicity and readability. It offers a large standard library and supports multiple programming paradigms, including object-oriented, imperative, and functional programming. Python is widely used for web development, data analysis, scientific computing, artificial intelligence, and more. Its clean and easy-to-learn syntax, along with a thriving community and extensive documentation, has made Python one of the most popular programming languages in the world.
Data analysis	Data analysis has been carried out via Python lenguage as well.

For manuscripts utilizing custom algorithms or software that are central to the research but not yet described in published literature, software must be made available to editors and reviewers. We strongly encourage code deposition in a community repository (e.g. GitHub). See the Nature Portfolio guidelines for submitting code & software for further information.

Data

Policy information about availability of data

All manuscripts must include a data availability statement. This statement should provide the following information, where applicable:

- Accession codes, unique identifiers, or web links for publicly available datasets
- A description of any restrictions on data availability
- For clinical datasets or third party data, please ensure that the statement adheres to our policy

Data are freely available at: https://zenodo.org/record/8430596

Research involving human participants, their data, or biological material

Policy information about studies with human participants or human data. See also policy information about sex, gender (identity/presentation), and sexual orientation and race, ethnicity and racism.

Reporting on sex and gender	Not applicable in our research
Reporting on race, ethnicity, or other socially relevant groupings	Not applicable in our research
Population characteristics	Not applicable in our research
Recruitment	Not applicable in our research
Ethics oversight	Not applicable in our research

Note that full information on the approval of the study protocol must also be provided in the manuscript.

Field-specific reporting

Please select the one below that is the best fit for your research. If you are not sure, read the appropriate sections before making your selection.

☐ Life sciences ☐ Behavioural & social sciences ☒ Ecological, evolutionary & environmental sciences

For a reference copy of the document with all sections, see nature.com/documents/nr-reporting-summary-flat.pdf

Ecological, evolutionary & environmental sciences study design

All studies must disclose on these points even when the disclosure is negative.

Study description	We proposed, implemented, and tested a synergistic approach between (deterministic) numerical weather prediction models and an AI-based strategy to predict lightning occurrence with unprecedented accuracy 2 days ahead.
Research sample	Not applicable
Sampling strategy	We employed k-fold cross-validation, a state-of-the-art technique, to address the challenge of ensuring the representativeness of the data sample used for model testing. This strategy is explained in detail in the Methods section.
Data collection	We use data from IMPACT ESP sensors used to detect lightning flashes in the LAMPINET detection network. These data are made available from the Italian Air Force Meteorological Service (Servizio Meteorologico dell'Aeronautica Militare). These data are freely available for research purposes upon request to the Italian Air Force Meteorological Service via email at dati.meteo@aeronautica.difesa.it. Observation period: 3 years, practically continuous in time.
Timing and spatial scale	Observation period: 3 years, practically continuous in time. Choice motivated by the concomitant availability of lightning prediction from HRES-ECMWF model, thus allowing a detailed comparison between our AI-based prediction and the deterministic predictions from HRES.
Data exclusions	No data were excluded.
Reproducibility	Not applicable: we did not carry out experiments but model prediction which are, by definition, reproducible.
Randomization	Not applicable
Blinding	Not applicable

Did the study involve field work? ☐ Yes ☒ No

Reporting for specific materials, systems and methods

We require information from authors about some types of materials, experimental systems and methods used in many studies. Here, indicate whether each material, system or method listed is relevant to your study. If you are not sure if a list item applies to your research, read the appropriate section before selecting a response.

Materials & experimental systems

n/a	Involved in the study
☒	☐ Antibodies
☒	☐ Eukaryotic cell lines
☒	☐ Palaeontology and archaeology
☒	☐ Animals and other organisms
☒	☐ Clinical data
☒	☐ Dual use research of concern
☒	☐ Plants

Methods

n/a	Involved in the study
☒	☐ ChIP-seq
☒	☐ Flow cytometry
☒	☐ MRI-based neuroimaging

Plants

Seed stocks	Not applicable in our research
Novel plant genotypes	Not applicable in our research
Authentication	Not applicable in our research
